# Association between *SOD1, CAT, GSHPX1* polymorphisms and the risk of inflammatory bowel disease in the Polish population

**DOI:** 10.18632/oncotarget.22675

**Published:** 2017-11-27

**Authors:** Malgorzata Mrowicka, Jerzy Mrowicki, Michal Mik, Radoslaw Wojtczak, Lukasz Dziki, Adam Dziki, Ireneusz Majsterek

**Affiliations:** ^1^ Department of Clinical Chemistry and Biochemistry, Medical University of Lodz, Hallera 1 Square, Lodz 90-647, Poland; ^2^ Chair of Surgery, Department of General and Colorectal Surgery, Medical University of Lodz, Hallera 1 Square, Lodz 90-647, Poland

**Keywords:** inflammatory bowel diseases, genetic polymorphism, antioxidant enzyme activity, oxidative stress

## Abstract

**Purpose:**

The main aim of this study was investigate the association between the genetic polymorphism of antioxidant enzyme genes: *SOD1, CAT* and *GSHPX1* and the risk of inflammatory bowel disease (IBD), including Crohn’s disease and ulcerative colitis in the Polish population.

**Methods:**

A total of 445 subjects including 200 patients with IBD and 245 controls were allowed in this study. We determined activity of superoxide dismutase (SOD), catalase (CAT), glutathione peroxidase (GPx1) and examination their association with the SNPs of respective genes (*SOD1* +35A/C, *CAT* C-262T and *GSHPX1* Pro197Leu). RFLP technique was used to determine the selected genes polymorphisms. Antioxidant enzymes activity were evaluated in erythrocyte hemolysate of 23 patients with non-active IBD and 30 healthy participants.

**Results:**

The A/C genotype and the C allele frequencies of A/C polymorphism of *SOD1* gene were significantly associated with the reduced risk of IBD (OR=0.43; 95% CI 0.23; 0.83). Alike, C/T (OR=0.45; 95% CI= 0.29; 0.70) and T/T genotype (OR=0.43; 95% CI= 0.21; 0.87) of *GSHPX1* gene polymorphism diminished the susceptibility to IBD. A significant decrease of CAT (*P*=0.028) and increase of GPx1 (*P*=0.025) enzyme activities were seen in IBD patients compared to control.

**Conclusions:**

Our data confirm dysregulated antioxidant capacity in patients suffering from IBD. Both, the *SOD1* A/C genotype as well as *GSHPX1* C/T and T/T genotypes may be associated with a reduction risk of IBD in the Polish population.

## INTRODUCTION

Literature presents that over 2.5 million Europeans and 1 million Americans suffering from inflammatory bowel disease (IBD) and the number of cases will increase [1]. IBD includes two common forms, Crohn’s disease (CD) and ulcerative colitis (UC), which are distinct chronic bowel-relapsing inflammatory disorders. IBD affects individuals of both sexes throughout life, and also is a risk factor for colorectal carcinoma. The exact cause of IBD is still unknown. It has been suggested that interactions between luminal microbial antigens and adjuvants, other undefined environmental exposures, the individual’s genetic susceptibility, and inappropriately sustained and severe autoimmune inflammatory responses are associated with the development of IBD [2].

Recently, oxidative stress has been proposed to be a factor that influences IBD. Amongst the immune regulatory factors, reactive oxygen species (ROS) are produced in abnormally high levels in IBD. Oxidative stress is a potential etiological and/or triggering factor for IBD, because the detrimental effects of free radicals have been well established in the inflammation process. The cause of the intestinal mucosa damage is not fully established, but the massively infiltrating polymorpho- and mononuclear phagocytic leukocytes are thought to contribute by producing large amounts of highly reactive transient chemical molecules, such as superoxide anion, hydrogen peroxide, the hydroxyl radical and hypochlorous acid. The cellular components of this inflammation are capable of producing nitric oxide (NO), besides ROS. Inducible nitric oxide synthase (iNOS) isoform produces much larger amounts of nitric oxide and is only expressed during inflammation, including IBD too. Thus, overproduction of ROS and reactive nitrogen species (RNS) leads to oxidative and nitrosative stress in the cells.

The first line of defense against ROS/RNS-mediated damage to constitutes system of antioxidant enzymes: superoxide dismutase (SOD1), catalase (CAT) and glutathione peroxidase (GPx1) that exists in all human cells. The differences in the regulation of expression between SOD1, CAT and GPx1 may not only reflect their importance in physiology, but may be also insufficient in remove of free radicals under inflammatory conditions such as IBD.

In our pilot analysis we had demonstrated that polymorphic variant of the *CAT -262* C/T may have protective effects in patients with UC [3]. Since the previous analysis of the patients in the group of half the smaller numbers did not make any clear conclusions, we decided to continue working and increase the number of IBD patients as well as the control group.

The aim of the present study was to analysis the relationship between the genetic polymorphisms of *SOD1, CAT and GSHPX1* and the prevalence of IBD, including Crohn’s disease and ulcerative colitis among the Polish population. Using the standard restriction fragment length polymorphism (RFLP) technique, we analyzed three SNPs of antioxidant enzyme genes: *SOD1* (rs2234694), *CAT* (rs1001179) and *GSHPX1* (rs1050450). Furthermore, we examined the activity of SOD1, CAT and GPx1 in red blood cells of IBD patients and controls and investigate their association with the SNPs of respective genes.

## RESULTS

### The association of *SOD1, CAT, GSHPX1* SNPs and the risk of IBD in a Polish population

The studied population consisted of 200 IBD patients as well as 245 controls. Patients and controls were subdivided according to their genotype for the +35A/C polymorphism of the *SOD1* gene, C-262T polymorphism of the *CAT* gene and Pro197Leu polymorphism of the *GSHPX1* gene. The genotype frequencies of the investigated polymorphisms are presented in Table 1. The statistcal power of our experiment was 100%.

**Table 1:** The genotype and allele frequency and odds ratios (OR) of the *SOD* A35C, *CAT* C-262T and *GSHPX1*Pro197Leu polymorphisms in patients with inflammatory bowel disease (IBD) and Crohn’s disease and ulcerative colitis compared to control group

	Controls n=245	IBD n=200	OR (95% CI) p-value	Crohn’s disease n=95	OR (95% CI) p-value	Ulcerative colitis n=95	OR (95% CI) p-value
*SOD* A35C genotype							
A/A	208 (0.85)	186 (0.93)	Ref.	89 (0.94)	Ref.	97 (0.92)	Ref.
A/C	36 (0.15)	14 (0.07)	**0.43(0.23-0.83) p=0.01**	6 (0.06)	**0.39(0.16-0.96) p=0.034**	8 (0.08)	0.48(0.21-1.06) p=0.066
C/C	1 (0.00)	0 (0.00)	-	0 (0.00)	-	0 (0.00)	-
allele							
A	452 (0.92)	386 (0.97)	Ref.	184 (0.97)	Ref.	202 (0.96)	Ref.
C	38 (0.08)	14 (0.04)	**0.43(0.23-0.81) p=0.007**	6 (0.03)	**0.39(0.16-0.93) p=0.029**	8 (0.04)	0.47(0.22-1.03) p=0.053
*CAT*C-262T genotype							
C/C	151 (0.62)	130 (0.65)	Ref.	59 (0.62)	Ref.	73 (0.70)	Ref.
C/T	79 (0.32)	59 (0.30)	0.85(0.38-1.92) p=0.699	30 (0.32)	0.97(0.57-1.63) p=0.92	27 (0.26)	0.71(0.42-1.19) p=0.188
T/T	15 (0.06)	11 (0.06)	0.98(0.42-2.29) p=1.000	6 (0.06)	1.02(0.38-2.76) p=1.000	5 (0.05)	0.69(0.24-1.97) p=0.484
allele							
C	381 (0.78)	319 (0.80)	Ref.	148 (0.78)	Ref.	173 (0.82)	Ref.
T	109 (0.22)	81 (0.20)	0.89 (0.64-1.23) p=0.471	42 (0.22)	0.99 (0.66-1.48) p=1.000	37 (0.18)	0.75(0.49-1.13) p=0.167
*GSHPX1*							
Pro197Leu genotype							
C/C	46 (0.19)^a)^	68 (0.34)	Ref.	33 (0.34)	Ref.	37 (0.36)	Ref.
C/T	172 (0.70)	115 (0.58)	**0.45(0.29-0.7) p=0.0004**	55 (0.57)	**0.46(0.26-0.77) p=0.003**	58 (0.56)	**0.42(0.25-0.71) p=0.001**
T/T	27 (0.11)	17 (0.09)	**0.43(0.21-0.87) p=0.018**	9 (0.09)	0.46(0.19-1.12) p=0.083	8 (0.08)	**0.37(0.15-0.91) p=0.027**
allele							
C	264 (0.54)	251 (0.63)	Ref.	121 (0.62)	Ref.	132 (0.64)	Ref.
T	226 (0.46)	149 (0.37)	**0.69(0.53-0.91) p=0.008**	73 (0.38)	**0.70(0.50-0.99) p=0.043**	74 (0.36)	**0.65(0.47-0.92) p=0.013**

^a^p<0.05 distribution not consistent with Hardy-Weinberg equilibrium

The A/C genotype and the C allele frequencies of A/C polymorphism of *SOD1* gene were associated with the reduced risk of IBD (OR=0.43; 95% CI 0.23; 0.83).

No statistically significant differences were found between both groups with regard to genotype distribution and allele frequencies of C-262T polymorphism of *CAT* gene.

Both C/T (OR=0.45; 95% CI 0.29; 0.70) and T/T genotype (OR=0.43; 95% CI 0.21; 0.87) of *GSHPX1* Pro197Leu gene polymorphism were associated with the reduced risk of IBD.

### The association of *SOD1, CAT, GSHPX1* SNPs and the risk of CD and UC in the Polish population

Table 1 show distributions genotype and allele frequencies in control group and patients with CD and UC for the polymorphic gene variant enzyme *SOD* +35A/C, *CAT* C-262T and *GSHPX1* Pro197Leu.

The A/C genotype of *SOD* gene polymorphism (OR = 0.39; 0.16-0.96) may had a protective effect in CD patients.

No statistically significant differences were found in UC and CD patients compared to control group in relation to genotype distribution and allele frequencies of C-262T polymorphism of *CAT* gene.

The C/T and T/T genotype of *GSHPX1* gene polymorphism may had a protective effect in UC patients. Also OR value for genotype C/T (OR = 0.46; 0.26-0.77) of *GSHPX1* indicates the protective effect in the CD patients.

### HWE test

The observed genotpype frequencies of *SOD1*+35A/C and *CAT* C-262T SNPs in the control subjects were consistent with Hardy-Weinberg equilibrium (HWE) (p=0.672; χ^2^= 0.18 and p=0.288; χ^2^=1.13, respectively), but the observed genotype frequences of the *GSHPX1*Pro197Leu SNP were not in agreement with HWE (p=0.00; χ^2^= 41.7), what suggests that polymorphic variant of the T allele associated with the disease risk may be eliminated from the population study.

### The level of erythrocyte antioxidant enzyme activity

Antioxidant enzyme : SOD1, CAT and GPx1 activity in erythrocyte hemolysates of patients with IBD in remission phase as well as control subjects are showed in Table 2. The study included 23 subjects in 2 groups. The group of patients with IBD in remission phase consisted of 10 women and 13 men who were aged 35.3±11.6 years. The control group, consisted of 30 healthy volunteers.

**Table 2:** The activity of superoxide dismutase (SOD1), catalase (CAT) and glutathione peroxidase (GPx1) in IBD patients and healthy subjects

	Control; n = 30	IBD; n = 23	*P-*value
SOD, U/gHb/100ml	2791.1 ± 509.0	2594.6 ± 426.3	*P*=0.142
CAT, BU/gHb	7.17 ± 1.79	6.21 ± 1.09^*^	*P*=0.028
GPx1, U/gHb	36.6 ± 8.9	43.4 ± 12.6^*^	*P*=0.025

^*^ *P* value considered statistically significant if < 0.05

Each value represents mean ± SD

The SOD1 (U/gHb/100ml) activity was not-significantly lower in the examined group of patients with IBD compared to controls (2594.59 ± 426.3 *vs*. 2791.1 ± 509.01; *P*=0.142). The statistically significant decrease in CAT (BU/gHb) activity was observed in erythrocyte of IBD cases relative to controls (6.21 ± 1.09 *vs*. 7.17 ± 1.79; *P=*0.028). The activity of GPx1 (U/gHb) was significantly increased in IBD patients versus healthy subjects (43.38 ± 12.6 *vs*. 36.56 ± 8.93; *P*=0.025).

Mean SOD1, CAT and GPx1 activity in IBD patients’ erythrocytes did not differ significantly with the SNPs of respective genes. The antioxidant enzyme activities depending on genotypes in patients with IBD are showed in Table 3.

**Table 3:** The antioxidant enzyme activities depending on genotypes in IBD patients: Statistical analysis

Genotype	Mean	SD	Max	Min	Median (25%; 75%)	Statistical analysis
***SOD***						
A/A	2653.8	425.8	3375.3	2014.6	2634.9(2306.2; 2981.8)	A/A vs. A/C *p*=0.523
A/C	2453.3	418.1	2999.5	1981.6	2416.1 (2189.3; 2717.4)	A/A vs. C/C *p*=0.235
C/C	2094.6	-	2094.6	2094.6	2094.6 (2094.6; 2094.6)	A/C vs. C/C *p*=0.800
***CAT***						
C/C	6.66	1.09	8.90	5.00	6.600 (6.13; 6.95)	C/C vs. C/T *p*= 0.050
C/T	5.63	0.90	6.70	3.90	6.650 (5.20; 6.35)	C/C vs. T/T *p*= 0.149
T/T	5.55	0.71	5.6	5. 05	5.55 (5.50; 5.60)	C/T vs. T/T *p*= 1.000
***GSHPX1***						
C/C	41.79	13.12	70.12	18.53	38.63 (34.24; 51.27)	C/C vs. C/T *p*= 0.190
C/T	50.11	11.90	68.42	32.98	47.52 (42.33; 58.63)	C/C vs. T/T *p*= 0.671
T/T	36.34	8.63	42.60	23.62	39.57 (31.16; 41.51)	C/T vs. T/T *p*= 0.073

## DISCUSSION

The aim of our study was to evaluate the role of *SOD1, CAT* and *GSHPX1* polymorphisms as the risk factors for IBD in the case-control studies. In the literature there are limited reports on the impact gene polymorphisms of antioxidant enzymes on IBD development.

In *SOD1*, the +35A/C polymorphism (dbSNP ID: rs2234694, MAF (C) = 0.016) is adjacent to the splicing point (exon3/intron3), being related to the SOD1 activity. This gene represents about 50-80% of the total SOD activity and is an important defense system against ROS [4]. In our study, we found that *SOD1* +35A/C polymorphism, the A/C genotype and the C allele frequencies was associated with protection against IBD in the Polish population. The potential protective effect with statistical differences was also observed for genotypes and alleles studied polymorphic variants of *SOD1* gene in CD patients. In the UC patients was not found statistical relationship with IBD but the observed protective tendency. These findings confirmed earlier our observations: trend protective, but statistically unrelated, it was observed for genotype T/T and T allele of the + 35A/C *SOD1* polymorphism in UC [3].

Kosaka et al. [5] by examining manganese superoxide dismutase *SOD2* Ala-9Val and NAD(P)H:quinone oxidoreductase 1 *NQO1* C609T gene polymorphisms have found that *SOD2* Ala-9Val genetic variations may influence on age of onset of ulcerative colitis while the *NQO1* C609T polymorphism may affect on severity of UC and steroid resistance in patients. Study Funke S. et al. [6] demonstrated no effect modification by tobacco smoke on the association between genetic polymorphisms in oxidative stress genes: catalase, manganese superoxide dismutase, myeloperoxidase, endothelial nitric oxide synthase and colorectal cancer risk. Despite the fact that smoking is considered to be initiator of colorectal tumorigenesis [7].

Three genetic polymorphisms have been reported in the promoter region of the *CAT* named A-21T (rs7943316), C-262T (rs1001179) and C-844T (rs769214). It has been indicated that erythrocyte catalase activity is significantly associated with the C-262T polymorphism (dbSNP ID: rs1001179, MAF (T) = 0. 126). Higher activity was showed for the CC genotype [8]. This variation alters the binding of transcription factors, and thus affects on the basal expression as well as blood catalase activity.

In a previous study we had demonstrated on a smaller number of patients that the polymorphism of antioxidant enzymes *CAT* gene C-262T may have protective effects in UC patients carrying the C/T variant [3].

In the present study, in comparisons of *CAT* enzyme polymorphism frequency between the CD and the UC and the control groups we found no statistically significant correlations. Despite this, our results indicate that participants carrying the C/T and T/T genotypes have lower CAT activity compared to C/C genotype. Based on expression studies, Ahn et al. [9] also reported that the C/C genotype was associated with elevated enzyme activity compared with variant C/T and T/T genotypes.

Our observation is not backed up by other investigators, since Khodayari S et al. [10] found that individuals heterozygous for the variant C/T genotype in the promoter region of the *CAT* C-262T polymorphism have higher risk of developing UC compared with C/C genotype. In separate study by Sousa VC et al. [11] the *CAT* C-262T polymorphism, alone or combined with the *GSHPX1* Pro197Leu, was associated with high risk of fibrosis severity of liver and hepatocellular carcinoma in subjects chronically infected with HCV. Moreover, Shen et al. [12] has demonstrated a significant association between the TT genotype of *CAT* C-262T and cancer risk in a meta-analysis including 9.777 cancer patients and 12.223 healthy participants. However, Chang D. et al. [13] did not find evidence to support an association between *CAT* A-21T polymorphism and colorectal cancer risk in Chinese patients.

The *GSHPX1* gene is located on chromosome 3. This gene contains polymorphism of the cytosine-to-thymine (C>T) substitution at codon 198 and 197. A C>T mutation at amino acid position 197 in *GSHPX11* changes proline (Pro) to leucine (Leu) and resulted in Pro198Leu and Pro197Leu variations (dbSNP ID: rs1050450, MAF (Leu) = 0.218). Thus, its substitution by other amino acids may change structural conformation of the active site region and modify the enzyme activity resulting in oxidant/antioxidant imbalance.

The results of our study suggested that *GSHPX1* Pro197Leu gene polymorphism had a possible a protective effect in IBD, both UC and CD patients. Our findings indicate that the C/T genotype was associated with the higher erythrocyte enzyme activity in patients with IBD, indicating that it could have a impact on risk IBD reduction. Thus the observed interaction between *GSHPX1* Pro197Leu and the risk of IBD might be caused by the complex interplay between smoking, alcohol consumption, supplementation of selenium trace elements, diet habits and GPx1 activity.

Contrary to our results, Pereira C.C. at al. [14] showed that the A/A genotype of *GSHPX1* polymorphism may associated with the risk of ulcerative collitis in the Portuguese population. Crawford A. et al. [15] reported that *GSHPX1* Pro/Leu genotype has been related to lung cancer, bladder cancer and diabetic T2DM polyneuropathy. Separate study reported a non-significant trend towards reduced lung cancer risk in 50-60 years old patients with the Leu allele of *GSHPX1* Pro/Leu polymorphism [15]. Hansen RD et al. [16] indicated that *GSHPX1* Pro198Leu, *hOGG1* Ser326Cys (8-oxoguanine glycosylase 1) and erythrocyte GPx1 enzyme activity were not associated with risk of colorectal cancer in the Danish population. It is interesting to note that authors determined statistically significant interaction between alcohol consumption and the *GSHPX1* Pro198Leu polymorphism in relation with colorectal cancer risk. Authors explain this interaction by an influence of alcohol on the GPx1 enzyme activity and emphasize the importance of lifestyle-related oxidative stress.

In patients with IBD in remission phase we found decreased activities of SOD (not significantly) and CAT (significantly), increased values of GPx1 enzyme (significantly) compared to the control group. Results our study suggested that patients with IBD may have an imbalance between ROS production and the antioxidant defense system due to colon mucosal injury (UC) or of any part of the digestive tract (CD).

Literature data indicate increased activity of SOD in patients with active intestinal inflammation, in contrast to patients with IBD in remission who had decreased values of enzyme activity. Our results are consistent with earlier research study [17, 18]. Whereas, GPx1 is a selenocysteine-containing enzyme and therefore the measurement levels of plasma selenium in IBD patients could explain our results - elevated GPx1 activity.

Several studies have shown decreased antioxidant enzyme activities and/or total antioxidant activity and increased ROS production in patients with IBD, including CD and UC. Maor I. et al. [19] reported that two lipid peroxidation products –malondialdehyde (MDA) and lipid peroxides in patients with CD were lower than control group. It is also interesting that the study have shown a higher activity of GPx1 and lower level of the antioxidant beta-carotene in serum of CD patients compared to control group. In contrast, Achitei D. et al. [20] revealed that active IBD patients had an increased SOD and GPx1 activity and high level of MDA versus control group. But, patients being in remission had decreased SOD and GPx1 activity and increased lipid peroxidation, compared to the control subjects. The authors explain that the low SOD and GPx1 in remission patients could be the consumption of antioxidants during the active phases. Whereas Boehm D. et al. [21] observed that patients with CD, particularly in active phase, had increased plasma lipid peroxidation products, as well as a lower levels of peroxidation potential and oxidative LDL.

Clinical studies indicated that oxidative stress during active CD depends on H_2_O_2_ production because immune peripheral cells of active CD patients had elevated SOD activity, H_2_O_2_ level and lipid peroxidation (MDA and 8-oxo-deoxyguanosine), inhibited CAT activity and mitochondrial function. The results suggested the significant role of mitochondria and oxidative stress in CD progress [22]. Aslan M. et al. [23] showed elevated peripheral DNA damage (measured using alkaline comet assay) and oxidative stress determinated using plasma total antioxidant capacity, total oxidant status and oxidative stress index in patients with UC. Authors reported that overproduction of free radicals, DNA damage were associated with reduced antioxidant levels and UC development.

In summary, our results provide some support for the *SOD1* A35C and *GSHPX1* Pro197Leu genotypes to may play a protective role in IBD susceptibility in the Polish population. Decreased activity of SOD (not statistically significant), CAT (statistically significant) and an increased activity of GPx1 (statistically significant) demonstrated an imbalance between ROS and cellular antioxidant defense systems. In the present study, antioxidant defense was assessed in the erythrocytes. Red blood cells are more exposed to oxidative stress than other cell types due to an abundance of heme iron and oxygen, which can generate superoxides, H_2_O_2_ and lipid peroxides. But to establish the actual impact of redox status imbalance on the progression of IBD, more correlation studies are necessary. Further studies are needed for assess the pro-antioxidant status in the colonic biopsies compared to erythrocytes of the patients with IBD.

## MATERIALS AND METHODS

### Study subjects

In the present study, 200 patients were included. The patients were divided into 2 groups: group I – 105 patients (62 men and 43 women; mean age 28±11.9) with ulcerative colitis (UC), group II –95 patients (45 men and 50 women; mean age 22±4.5) with Crohn’s disease (CD). The patients were hospitalised in the Department of General and Colorectal Surgery, Medical University of Lodz. UC and CD were diagnosed on the basis of radiological, pathological and clinical criteria. The control group consisted of 245 healthy people (165 men and 80 women; mean age 44±4.3) without symptoms of the intestinal mucosa.

All subjects involved in the study went through detailed anamnestic questionnaire, medical history, physical examination and standard laboratory analysis. The study excluded subjects with disease such as infectious diseases and/or systemic infections, diabetes mellitus, chronic kidney disease, rheumatoid arthritis, chronic hepatitis. Because these disease have been associated with *SOD* and other antioxidant enzymes polymorphisms [11, 24, 25, 26]. Patients who use of anti-inflammatory medications for example: angiotensin converting enzyme inhibitors, statins or β-sympatholytic drugs, oral coticosteroids, aminosalicylic acid, non-steroidal anti-inflammatory agents and/or antioxidant supplements that may affect on the activity of antioxidant enzymes were excluded.

We examined the group of 445 individuals who gave free consent to participate in the study.

Research project has been approved by Bioethics Committee of the Medical University of Lodz number RNN/835/09/KB.

### DNA analysis

DNA was extracted from blood samples using the Genomic Blood Mini Ax in accordance with the manufacturer’s instructions.

Analysis of polymorphic variants of antioxidant enzymes *SOD + 35A/C, CAT -262C/T, GSHPX1 Pro197Leu* was performed using PCR-RFLP. The PCR reaction was performed in a Multi-Gene thermocycler (Labnet International Inc) in a total volume of 15 μl, containing 100 ng of genomic DNA, primer, polymerase mix and water in a total volume of 15 μl. After amplification of PCR products and after 16 hours of digestion by restrictive enzyme (HhaI for *SOD*, Smal for CAT, ApaI for *GSHPX1)* the samples were separated by electrophoresis on a 2% agarose gel and fragments obtained restrictive were visualize by staining with ethidium bromide.

### Blood samples and enzyme assays

Blood samples were collected from patients and controls in the morning, before breakfast. Erythrocytes were separated from blood plasma by centrifugation (10 min, 710 g) at 4°C and washed 3 times with 0.9 % NaCl before examination. After the supernatant was removed, rinsed erythrocytes were hemolyzed by adding distilled water ana partes aequales and then freezes at the temperature of -18°C. The hemolysate, after being defrosted was used in measurement of enzyme activities.

SOD1 activity was measured according to procedure by Misra and Fridovich (Misra and Fridovich, 1972) and expressed in adrenaline units (U/g Hb/100 ml). The activity was determined by the absorbance increase at 480 nm with a spectrometer following by the auto-oxidation of epinephrine inhibited by SOD.

CAT activity was determined according to spectrophotometric procedure by Beers and Sizer (Beers and Sizer, 1952) and calculated as Bergmeyer units (BU/g Hb). The method is based on measured enzyme activity by recording H_2_O_2_ decomposition at 240 nm with a spectrometer.

GPx1 activity was assayed by the spectrophotometric procedure described by Little and O’Brian (Little and O’Brian, 1968) and presented as enzymatic units (U/g Hb). The difference in the rate of GPx reaction with glutathione and kumen in the sample was used for its activity determination by absorbance measurement at 412 nm with a spectrometer.

For enzyme activities determination UV/VIS spectrophotometer Lambda 14P (Perkin Elmer, USA) was used.

Hemoglobin (Hb) concentration in erythrocyte hemolysate for the calculation of enzyme activities was estimated after conversion into cyanmethemoglobin with Drabkin’s reagent (Aqua-Med, Poland) (Van Kampen and Zijlstra, 1961).

### Statistical analysis

The activity of enzymes was expressed as mean value ± SD. The differences between means was evaluated by applying the Student’s *t*-test. The Mann-Whitney test was used to correlate between antioxidant enzymes activity and enzymes genotypes in IBD patients.

Each polymorphism was tested to ensure that it did not deviate from Hardy-Weinberg equilibrium by the χ^2^ test. Odds ratio (OR) and corresponding 95% confidence intervals (CI) were used to assess correlations between genotypes and alleles and IBD. Analyses were performed using STATISTICA 6.0 package (Statsoft, Tulsa, OK, USA).
